# Khat use as risk factor for psychotic disorders: A cross-sectional and case-control study in Somalia

**DOI:** 10.1186/1741-7015-3-5

**Published:** 2005-02-12

**Authors:** Michael Odenwald, Frank Neuner, Maggie Schauer, Thomas Elbert, Claudia Catani, Birke Lingenfelder, Harald Hinkel, Heinz Häfner, Brigitte Rockstroh

**Affiliations:** 1 Department of Psychology, University of Konstanz, Fach D25, D-78476 Konstanz, Germany; 2 Outpatient Clinic for Refugees, University of Konstanz, Feursteinstr. 55, Haus 22, D-78479 Reichenau, Germany; 3 Ctr. for Psychiatry Reichenau (ZPR), Feursteinstr.55, D-78479 Reichenau, Germany; 4 Worldbank, Multi-Country Demobilization and Reintegration Program in the greater Great Lakes Region of Africa, Goma, Democratic Republic of Congo; 5 AG Schizophrenieforschung, Zentralinstitut für Seelische Gesundheit, J5, D-68159 Mannheim, Germany

## Abstract

**Background:**

Little is known about the prevalence of khat-induced psychotic disorders in East African countries, where the chewing of khat leaves is common. Its main psycho-active component cathinone produces effects similar to those of amphetamine. We aimed to explore the prevalence of psychotic disorders among the general population and the association between khat use and psychotic symptoms.

**Methods:**

In an epidemiological household assessment in the city of Hargeisa, North-West Somalia, trained local interviewers screened 4,854 randomly selected persons from among the general population for disability due to severe mental problems. The identified cases were interviewed based on a structured interview and compared to healthy matched controls. Psychotic symptoms were assessed using the items of the WHO Composite International Diagnostic Interview and quantified with the Positive and Negative Symptoms Scale. Statistical testing included Student's t-test and ANOVA.

**Results:**

Local interviewers found that rates of severe disability due to mental disorders were 8.4% among males (above the age of 12) and differed according to war experiences (no war experience: 3.2%; civilian war survivors: 8.0%; ex-combatants: 15.9%). The clinical interview verified that in 83% of positive screening cases psychotic symptoms were the most prominent manifestations of psychiatric illness. On average, cases with psychotic symptoms had started to use khat earlier in life than matched controls and had been using khat 8.6 years before positive symptoms emerged. In most cases with psychotic symptoms, a pattern of binge use (> two 'bundles' per day) preceded the onset of psychotic symptoms, in contrast to controls of the same age. We found significant correlations between variables of khat consumption and clinical scales (0.35 to 0.50; p < 0.05), and between the age of onset of khat chewing and symptom onset (0.70; p <0.001).

**Conclusion:**

Evidence indicates a relationship between the consumption of khat and the onset of psychotic symptoms among the male population, whereby not the khat intake *per se *but rather early onset and excessive khat chewing seemed to be related to psychotic symptoms. The khat problem must be addressed by means other than prohibition, given the widespread use and its role in Somali culture.

## Background

The present study investigated the relationship between psychotic symptoms and the abuse of khat in the Horn of Africa. The leaves of the khat shrub (*catha edulis*) are traditionally chewed in Arab countries, the Horn of Africa and East Africa [1] and recently this habit has spread to Western countries including the USA [2]. Due to improved transportation facilities, khat consumption has substantially increased during recent decades. This is reflected in the most recent issue of the World Drug Report: in 2001 five countries reported an increase in khat use and none a decrease; in 2002 an increase was reported in four, a decrease, again, in none [3]. Kalix (1996) [4] estimates that about 6 million individual portions are consumed each day worldwide. During our field work in the city of Hargeisa, North-West Somalia (Somaliland), where khat use is not restricted by law, we observed that current ways of intake do not correspond to the documented traditional use in the region. The traditional way of consumption was socially highly regulated: adult males (more seldom females) would gather and chew khat together at a so-called 'khat party', usually on weekends and afternoons until the time of the evening prayer [5,6]. Contrary to this formerly restricted use, current habits involve use by adolescents, chewing khat in tea-shops that operate day and night, early morning use, as well as "binging" and "speed runs" that may last for more than 24 hours. Our study was based on observations by social workers of collaborating non-governmental organizations and by our team during field studies on war-related trauma. It was shown that the widespread use of khat is related to the large number of individuals with visible signs of psychosis who are either homeless or kept in hiding, e.g. in physical chains, by family members who afraid to expose them to the general public.

The main psycho-active component of khat leaves is cathinone (S(-)alpha-aminopropiophenone) [7]. Cathinone resembles amphetamine in chemical structure and affects the central and peripheral nervous system [8,9] and behavior [10,11] similarly. Amphetamines and some of its derivatives have been shown to induce psychotic symptoms in experimental settings in humans [12,13] and animals [14] and have been known to exacerbate psychotic states in psychiatric patients [15,16]. Similarly, khat-induced psychotic states have been described in several case studies [17-20]. However, the number of group, community and population-based studies on khat use and psychiatric symptoms [21-23] is still limited. Despite the ongoing scientific debate about amphetamine-induced psychosis it remains unclear whether the use of amphetamine-like substances including khat may actually *cause *a psychotic disorder in an otherwise healthy individual, or *trigger *the onset of schizophrenia in an individual with high vulnerability to the disease [24,25]. The fact that presumed amphetamine psychoses do not fully remit within weeks of abstinence in a substantial percentage of individuals [26] may also suggest that those individuals actually had a amphetamine-triggered schizophrenia. Increased drug use among psychotic patients may also come from an attempt to counteract nonspecific physical symptoms or side effects of neuroleptics [27].

The first goal of this study was to verify the impression of an unusually high prevalence of psychotic disorders in the city of Hargeisa via an epidemiological survey carried out in a representative sample of households. In addition, we wished to study the association between khat abuse and psychotic disorders. If khat abuse does induce psychotic disorders, a higher prevalence of psychotic diagnoses, mainly in men (as women rarely consume khat), was to be expected in Hargeisa compared to localities with less khat use. In addition, a case-control study served to examine whether individuals identified in the survey suffering from psychotic symptoms presented a pattern of khat consumption different from matched controls.

## Methods

### Sample

In order to screen for households with mentally ill members, 612 households with 4,854 members were randomly selected as representative of the city of Hargeisa (approximately 400,000 inhabitants). For household selection the town was first subdivided into 30 sections of approximately equal populations. For this purpose we used the same sections as UNICEF in their assessment of vaccination coverage for children in October 2002, which were defined in a joint approach by UNICEF and the municipality of Hargeisa. A section had to be subdivided into square-shaped clusters if it was L-shaped, had a natural boundary within its limits (e.g. a steep hill) or had a much greater length than depth. A map for each section or cluster was produced using an aerial photograph. For random selection the geographical center of each cluster was determined and a random direction was chosen using a compass, a watch and a list of random numbers between 1 and 12 (a watch was oriented according to the compass, whereby 12 o'clock was adjusted to North; the random number defined the direction according to the hours on the face of the clock). Following the random direction until the border of the cluster was reached, all houses on the right side within a range of 15 meters were numbered. The first household to be approached was selected by a second set of random numbers. The next houses were selected by door-to-door distance until the predefined number was reached. Trained local staff interviewed the heads of the 612 households. If the head of household was not available, another adult member of the household was asked to answer to the questions. If no household member could be interviewed, the next house was selected according to the selection procedure.

The following question was used to determine whether a person severely disabled due to mental health problems resided in the household: "Are there any members of your household who currently have mental problems that are so severe that the person has been unable to provide income or has been unable to help in the household for at least four weeks?" This criterion was fulfilled for 169 (137 male, 32 female) cases. These individuals will be referred to as 'positive screening cases'. A subsample of 52 'positive screening cases' was then randomly selected and examined in a clinical interview. In this group there were 44 males and 8 females. These individuals will be referred to as 'examined cases'.

For each 'examined case' a matched control, who did not meet the criteria for disability due to mental health problems, was identified. Controls were matched by gender, age, and educational level. These 'case controls' were subjected to the same clinical interview as the examined cases. Forty-nine of the 52 examined cases and one control were diagnosed with psychiatric or neurological disorders (on this basis we estimated a sensitivity of 0.98 and a specificity of 0.94 for the screening procedure). We included only those forty-three examined cases (82.7%) in the further analysis who – in addition to the impairment of function – showed as main manifestations of mental problems at least one very severe or two moderate positive psychotic symptoms in the absence of organic somatic damages that might explain them; from hereon these are termed 'cases with psychotic symptoms'. The disorders of other examined cases were stroke (2), traumatic brain injury (1), mental retardation (2), and dementia (1). The case control with a positive diagnosis reported hallucinations with intact reality testing during khat intoxication and was replaced by a healthy individual. Socio-demographic characteristics for the two studied groups are summarized in Tables 1 and 2.

**Table 1:** Socio-demographic characteristics of the sample of N = 4,854. For socio-demographic data, mean ± standard deviation and percentages and absolute numbers (in parenthesis) respectively are reported. P-values refer to differences between ex-combatants, civilian war survivors, and persons without any war experience tested by Kruskal-Wallis^1 ^and chi ^2 ^^2^.

	**Whole group**	**Ex-combatants**	**Civilian war survivors**	**No war experience**	**p**
Total Number	4,854	250	2,667	1,937	-
Male	2,449	240	1,201	1,008	
Female	2,405	10	1,466	929	< 0.001^2^
Age	22.2 ± 17.8	42.8 ± 13.5	28.7 ± 16.6	10.7 ± 12.1	< 0.001^1^
Age range	0 – 102	12 – 102	0 – 101	0 – 90	-
Years of formal education	2.6 ± 4.1	5.2 ± 5.6	3.3 ± 4.3	1.4 ± 3.0	< 0.001^1^
Economic situation of household*	2.8 ± 1.8	2.5 ± 1.7	2.9 ± 1.7	2.8 ± 1.8	0.005^1^
Percentage of khat users in week before interview among men > 12 yrs	31.3% (495 of 1,581)	60.3% (144 of 239)	28.1% (306 of 1,090)	17.9% (45 of 252)	< 0.001^2^
Khat bundles/week before interview among khat chewing men > 12 yrs	7.6 ± 4.4	7.9 ± 4.8	7.6 ± 4.4	6.7 ± 3.2	0.544^1^
'Screening cases' among men > 12 yrs	8.4% (133 of 1,581)	15.9% (38 of 239)	8.0% (87 of 1,090)	3.2% (8 of 252)	< 0.001^2^
'Screening cases' among women > 12 yrs	1.9% (30 of 1,600)	20% (1 of 5)	1.6% (22 of 1,372)	2.8% (7 of 248)	0.066^2^

* The household economic situation was approximated as the sum of four significant assets (water tap, electrical power, TV set, car) and type of housing (hut = 1, house = 2, closed compound = 3). The mean of the two ratings is presented. For the combined rating Cronbach's Alpha = 0.78; correlation with income = 0.73, p < 0.001.

**Table 2:** Socio-demographic characteristics of the 43 cases with psychotic symptoms and 43 'case controls.' Mean ± standard deviation and percentages and absolute numbers (in parenthesis) respectively are reported. P-values refer to group differences assessed by t-test/Wilcoxon test.

	**Patients with psychotic symptoms**			**Matched controls**			**Sig.**
	
	**total**	**male**	**female**	**total**	**male**	**female**	**p**
N	**43**	38	5	**43**	38	5	**1.0**
age	**33.9 ± 10.5**	34.6 ± 10.0	28.6 ± 13.8	**34.7 ± 10.3**	35.3 ± 10.0	30.4 ± 12.8	**0.718**
Education (yrs in school)	**5.6 ± 4.5**	6.4 ± 4.3	0.0	**6.3 ± 4.9**	7.1 ± 4.6	0.0	**0.511**
Martial status (% married)	**16.3% (7)**	18.4 % (7)	0 % (0)	**44.2% (19)**	50.0% (19)	0% (0)	**0.005**
Employed or at school	**0% (0)**	0% (0)	0% (0)	**41.9% (18)**	47.4% (18)	0% (0)	**< 0.001**

### Procedures and materials

All interviews were carried out in October and November 2002. Prior to the interview, participants were informed about the background of the study and the survey procedure. All participants signed a written informed consent, which was first read and explained to them (as most of the participants were illiterate). In the case of mentally challenged participants, background and procedure were also explained to the responsible caretakers whose written informed consent was a prerequisite for their participation. Interviews were approved by the local authorities and the National Demobilization Committee of Somaliland. Representatives of the different communities were informed and invited to observe the assessment in the field. Newspaper advertisements, daily radio programs and flyers informed the population about and helped them to understand the real purpose of the assessment (at first rumors had spread that the research team would provide free medication for mentally disturbed individuals and created unreal expectations). Consequently, the level of cooperation was extremely high. Out of 73 households approached under the supervision of the first author only three refused their participation. Therefore, we estimate that in total less than 5% of all households refused participation.

Local interviewers were recruited among NGO and hospital staff who already had experience in working with mentally disabled persons. They participated in a two-week training course, which involved teaching the basic psychiatric concepts (e.g. psychotic symptoms), interviewing skills, training on the screening-instrument and field work under close supervision of experts. After the end of the course, fourteen of the seventeen trainees were employed for the duration of the study. Five interviewer teams, each comprising one male and one female interviewer, and four local supervisors did the field work. The local supervisors received additional training on the random sampling method. During the first weeks of interviewing the first author closely supervised the teams in the field.

The screening interview assessed individual socio-demographic information, khat consumption and experiences in the civil war (subjects were grouped as either active war participants, civilian war survivors, or without any war experiences). Khat consumption (number of bundles/week) was assessed for the week prior to the interview. The household economic situation was approximated as the sum of four significant assets (water tap, electrical power, TV set, car) and type of housing (Table 1).

Clinical interviews were carried out by some of the authors (MO, MS, CC, BL), all trained in the assessment of psychotic disorders and PANSS rating. Socio-demographic information and war-history were detailed. A standard event list was used to quantify the number of traumatic event types a person had experienced; the list included 11 types of events: active combat experience, accident, natural disaster, witness murder or killing, rape, sexual molestation, physical assault, being kidnapped or captured, torture, other, or suffered shock because a close person had experienced a traumatic event. In a short semi-structured qualitative interview the main areas of psychological condition and functioning were assessed. Additionally, psychotic symptoms were assessed using the items of the Composite International Diagnostic Interview (CIDI), Section G [28]. The individual's age at which the family first noticed positive psychotic symptoms that prevailed over six months was taken as indication of the onset of a psychotic disorder. Khat consumption (average number of bundles/day) was assessed for the week before the interview and for the week prior to the onset of symptoms in examined cases. Case controls were asked about consumption in the week prior to the interview and for consumption at the age of symptom onset reported by his/her examined case-pair (Table 2). As many examined cases were not able to give valid information, the primary care taker and other family members were also interviewed.

Clinical interviews were administered in the English language with the help of trained local interpreters, who had participated in the same training course as the interviewers. After the interview, the interviewing psychologist rated the current psychopathology using the Positive and Negative Syndrome Scale, PANSS [29].

All items of the standardized interviews were translated from English into Af Somali by mixed teams of clinical experts and bilingual staff. Several steps of independent back-translations and corrections were necessary until a satisfactory translation was achieved. Content validity was assured by the involvement of clinical experts at all levels of the translation process. Also, interpreters were introduced to the concepts targeted by the respective questions.

### Data analysis

Differences between cases and controls were confirmed using chi^2^-tests (or Fisher's exact tests), binomial testing, Student's t-test or Wilcoxon, and ANOVA or Krsucal-Wallis test (all tests two-tailed). Univariate Analysis of Variance (ANOVA) was used to explore the effects of war-trauma on the khat intake. Means and standard deviations are reported.

## Results

Of the sample of 4,584 inhabitants of Hargeisa the screening interview disclosed 169 positive screening cases (i.e. 3.5%). Positive screening cases were more often male than female (133 of 1,581 men, i.e. 8.4%, and 30 of 1,600 women above the age of twelve years, i.e. 1.9%; p < 0.001; Table 1). Khat chewing was more frequent among male positive than among negative screening cases above the age of 12 years: 46.6% of the 133 positive screening cases had chewed khat in the week before the interview in contrast to 29.9% of the 1,448 negative screening cases (p < 0.001). Consumers among positive screening cases had also chewed a greater amount of khat in the week preceding the interview (positive screening cases: 4.1 ± 6.3 bundles; negative screening cases: 2.2 ± 4.0 bundles; p = 0.001).

The proportion of positive screening cases was substantially higher among males above the age of 12 who had active war experience (ex-combatants) than in male civilian war survivors of the same age (p < 0.001, Table 1). The latter proportion was significantly higher than in men without any war experience (p = 0.007).

Psychotic symptoms meeting our criteria were determined for 83% (43) of the examined cases. Retrospective investigation suggested that the onset of psychotic signs occurred at the age of 23.4 ± 9.9 years (N = 38 men: 24.1 ± 9.8 years; N = 5 women: 18.4 ± 9.6 years; p = 0.230).

PANSS ratings of these 43 cases showed a substantially higher magnitude of current psychotic symptoms compared to a sample of 240 medicated schizophrenic patients [30] in the following subscales: Positive 27.1 ± 7.3, Negative 32.0 ± 8.9, Composite -4.9 ± 10.6, General Psychopathology 52.7 ± 12.7, Anergia 16.6 ± 5.5, Thought Disturbance 16.4 ± 5.3, Activation 7.8 ± 3.0, Paranoia/Belligerance 10.8 ± 4.1, Depression 9.5 ± 3.9, and Supplemental Scale 17.4 ± 7.4. In further exploratory observations, we noticed a high tendency towards aggressive and hyperactive behavior. During interviews, most patients reported that they were in contact with a ghost ('djin'), often associated with auditory, visual and somatosensory hallucinations.

Fifteen of the 43 cases with psychotic symptoms (35%) were under current medication at the time of assessment; another nine (21%) had received medication in the past. A variety of drugs had been given, ranging from neuroleptics (12 patients) to prometazine (6), benzodiazepines (3), tricyclic antidepressants (3), carbamazepine (1) and other unknown drugs (10).

Uncontrollable (disruptive, violent) behavior had led to restraint of cases with psychotic symptoms by chaining them to an object in 28 of the 38 men (i.e. 74%) and 3 of the 5 women (i.e. 60%) (p = 0.608). Additionally, 9 men (24%) and 3 women (60%) had been locked up to control their behavior (p = 0.589). On average, the 31 cases with psychotic symptoms who had ever been chained had spent 4.2 ± 5.2 years in chains (men: 4.5 ± 5.4 years; women: 1.8 ± 1.9 years; p = 0.410) and the 10 cases who had ever been locked up spent on average additional 5.2 ± 6.5 years restrained (men: 4.0 ± 6.6 years; women: 10.0 ± 4.2 years; p = 0.264).

**Figure 2:**
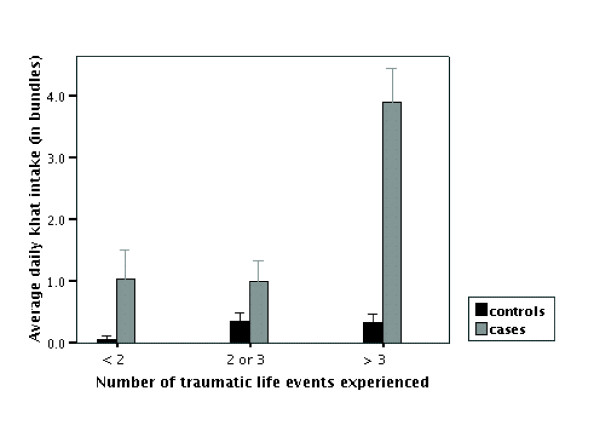
**Khat intake and traumatic experiences. **Average daily khat intake in bundles (at the age when the case with psychotic symptoms showed onset of symptoms) according to number of traumatic life events. We divided the cases with psychotic symptoms and case controls into three groups according to the number of event types experienced as follows: none or one: 11 cases and 13 controls; two or three: 9 cases and 15 controls; more than three: 11 cases and 14 controls. Bars indicate mean and standard error.

The proportion of khat user was higher among cases with psychotic symptoms than among matched controls: all except one of the 38 male psychotic cases, in contrast to 25 of the 38 male controls, had used khat (p < 0.001). In the cases with psychotic symptoms, regular khat consumption had started at an earlier age (16.6 ± 4.8 years) than among their matched controls (20.7 ± 7.0 years, p = 0.010; Figure 2). All except one case with psychotic symptoms (compared to 61% of controls, i.e. 14 of 23) had started to chew before the age of 23 years (p = 0.004). None of the women admitted to having ever chewed.

**Figure 1:**
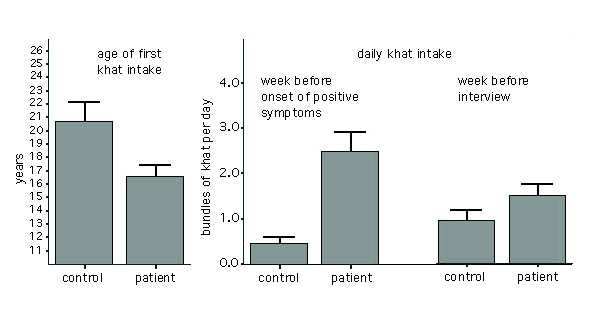
**Patterns of khat consumption. **Left: age of first khat intake among lifetime chewers in patients with psychotic symptoms (35) and case controls (23); right: amount of khat use (in 'bundles' per day) in the week before the onset of first positive symptoms (25 cases with psychotic symptoms, 24 controls) and in the week before the clinical interview (16 cases with psychotic symptoms, 9 controls). Bars indicate mean and standard error.

In the weeks preceding the onset of psychotic symptoms, cases with psychotic symptoms had chewed an average of 2.5 ± 2.0 bundles/day compared to 0.5 ± 0.6 bundles/day in controls (p < 0.001; Figure 1). Excessive khat intake (> two bundles/day) in this period was found in 78% of chewers among male cases with psychotic symptoms (i.e. 29 of 37) but in only 4% of chewers among controls (i.e. 1 of 25; p < 0.001). For the cases with psychotic symptoms, the age of onset of khat chewing correlated significantly with symptom onset (r = 0.70, p < 0.001). The lapse of time between first use of khat and onset of symptoms was greater than one year in 31 of the 38 male cases with psychotic symptoms (i.e. 82%); it varied around a mean of 8.6 ± 6.6 years (median 7 yrs). In the week before the interview 54% of the lifetime khat chewers among male cases with psychotic symptoms (i.e. 20 of 37) and 36% of the controls (i.e. 9 of 25) had chewed khat (p = 0.162). Furthermore, psychotic patients consumed an average of 1.5 ± 1.0 bundles/day, with controls consuming an average of 0.9 ± 0.7 (p = 0.172; Figure 1).

Khat use correlated with severity of symptoms: In cases with psychotic symptoms onset of khat use correlated significantly with higher scores on the PANSS subscales Negative Symptoms (r = - 0.36, p = 0.039, N = 34), General Psychopathology (r = - 0.44, p = 0.009; N = 34), Anergia (r = - 0.39, p = 0.022, N = 34) and Thought Disorder (r = - 0.42, p = 0.014, N = 34), while the amount of khat consumed prior to onset of psychotic symptoms correlated significantly with the subscale Paranoia/Belligerence (r = 0.49, p = 0.006, N = 30) and the Supplemental scale (r = 0.50, p = 0.005, N = 30). The amount of khat consumed in the week before the interview correlated with the PANSS subscale Anergia (r = - 0.35, p= 0.029, N = 38).

The number of traumatic event types experienced did not differ between cases with psychotic symptoms and matched controls (cases with psychotic symptoms: 3.1 ± 2.3 events; matched controls: 2.9 ± 2.4 events; p = 0.749). Also, the age when the first traumatic event was experienced did not differ between them (cases with psychotic symptoms: 20.2 ± 9.6 years; matched controls: 20.6 ± 9.2 years; p = 0.853). In order to estimate the relationship between number of experienced traumatic events and khat intake we used univariate ANOVA with khat intake before onset of psychotic symptoms (for controls at the age of symptom onset of matched pair) as dependent variable, and number of traumatic events in three categories (no or one event, two or three, four or more) and being a case or a control as independent variable. We found significant effects for the number of traumatic events (Sum of squares 36.6; df 2; F = 17.028; p < 0.001) and for the variable 'case or control' (Sum of squares 53.9; df 1; F = 50.136; p. < 0.001) and a significant effect of their interaction (Sum of squares 31.0; df 2; F = 14.404; p < 0.001) (total R^2 ^= 0.620). In Figure 2 the results are displayed graphically.

## Discussion

The present study revealed a number of interesting and relevant findings.

(1) In a representative subsample of male inhabitants of Hargeisa (older than 12 years) we found 8.4% severely disabled due to mental problems; of these, 83% had severe psychotic symptoms. Compared to the prevalence rates of psychotic disorders reported for male samples elsewhere [31,32] this is higher than expected. Furthermore the gender ratio among the mostly psychotic positive screening cases was unusual (1 women to 7.7 men).

(2) Khat abuse showed a significantly earlier onset and was more frequently excessive in male cases displaying psychotic symptoms than in matched controls.

(3) There is a higher vulnerability to disability due to mental disorders in those groups of society who were directly involved in combat in the past.

(4) Among the positive screening cases examined and their matched controls we found that khat intake before the onset of psychotic symptoms (respectively at the same age for matched controls) was higher when more traumatic events had previously been experienced.

These results are intriguing, as we may have detected only the very tip of the iceberg with our sub-optimal screening procedure, leaving less severe cases of mental and neurological disorders undiscovered.

The findings of the present study suggest that there is an association between khat consumption and psychosis; however the existence of a causative relationship and its direction between the two remains unclear. Furthermore, it seems that it is not the consumption of khat *per se *but rather specific patterns of it that are related to psychosis, especially early onset in life and the excessive intake (> two bundles per day). Both seem to be related to active participation in war, e.g. child ex-combatants used khat while fighting in order to counteract fear and enhance performance [33]. Excessive khat consumption has already been reported to be associated with higher severity of psychiatric symptoms [21]. Our data also suggest that there might be a moderating effect of the number of traumatic experiences on subsequent khat intake. This would be in line with the self-medication hypothesis.

Khat consumption might be related to the outbreak of psychosis in various ways. Considering psychoses as the result of genetic and acquired vulnerability and additional stress factors, the early onset of khat abuse may have substantially increased the risk in already vulnerable individuals. In animal studies, damage imposed on the developing brain, e.g. by drugs, increases the potential of amphetamine-like agents to change neuro-chemical systems and to induce psychotic-like behavior [34]. In humans, drug abuse during puberty has been found to precede the onset of psychotic symptoms and prodromi [35] and to be related to poorer treatment outcome [36]. Additional risk factors and particular stressors, such as war experiences, may contribute to an increased vulnerability. It is presently unclear, however, whether traumatic experiences act indirectly through higher drug consumption in traumatized men or whether they exert a direct effect on the brain, increasing the risk of developing psychosis. The temporarily higher khat abuse preceding first symptoms in cases with psychotic symptoms compared to case-controls may suggest that khat consumption is the primary agent, causing the onset of psychoses. This assumption is further strengthened by the inequality in the gender ratio, as women are generally denied access to the drug.

Moreover, khat consumption may affect the course of a psychotic disorder and the maintenance of symptoms. In contrast to the high khat consumption prior to the onset of psychotic symptoms, the amount of current khat consumption did not differ between cases with psychotic symptoms and controls and a significant number of patients did not have current access to the drug. Drug-effects on the course of illness may be attributed to sensitization [37] or to lasting neurotoxic effects of prolonged stimulant intake [14]. To clarify the question of how continued khat consumption might affect the further development of psychotic symptoms, the number of relapses related to khat intake would be of tremendous importance. Our observation was that once a patient has developed severe psychotic symptoms he is restrained (e.g. chained) and kept away from the drug until symptoms remit. However, as soon as he is released the patient engages again in (often excessive) khat consumption until symptoms relapse and he is restrained again. Many patients had lived through this circle several times. In Western samples of amphetamine-induced psychosis, first relapses have mostly been studied [37]. Furthermore, it seems plausible that the conditions under which cases with psychotic symptoms were frequently kept may have infuenced the course of any psychopathological development.

Some methodological shortcomings of the study are related to the nature of field work in this specific post-conflict setting, which involved restricted freedom of movement due to the security situation and limited resources. However, although a perfect design is not possible in a rather complex field situation, the importance of such studies is evident from the fact that little or no information from post-war Somalia is available. First, we could not control whether our sample was comparable to the population from which it was drawn due to the lack of recent statistics in Somalia.

Second, the validity of our screening and clinical interview can be questioned because of various points, e.g. whether the descriptions of symptoms and disability might be adequate for the Somali culture, or whether factors such as interpretation or the interviewing of a whole family rather than a single patient might distort the information. In our approach we decided to use disability as a selection criterion and a descriptive rather than a normative approach to identify the reasons for it. Thus, we decided to use the more unspecific terms 'cases with psychotic symptoms' or 'psychotic symptoms' rather than 'schizophrenia' or 'drug-induced psychosis'. However, the fact that our local interviewers found 123 of the 169 positive screening cases (72.8%) restrained, i.e. chained or locked up, and the overall high average PANSS scores of the examined cases, show that there are 'hard' criteria, which justify the use of the term 'psychosis' and validate our screening. Nevertheless, we recognize that with our method we must have failed to discover persons suffering from psychotic and other mental disorders (false positives). But assuming that this error is somehow the same for the whole sample, our finding that the group of society that engages most in khat chewing (males above the age of 12) most often suffers severe impairment due to mental disorders shows evidence that khat is related to mental health problems.

Thirdly, the fact that we could not choose our matched controls from the pool of negative screening cases – the resources that would have been necessary to (repeatedly) contact and arrange appointments with them exceeded our capacities – leads without any doubt to an overestimation of the specifity of our detection procedure.

Furthermore, the retrospective third-person assessment of the onset of psychotic signs, as well as the retrospective and subjective assessment of khat use, will have introduced some error variance in the data. However, we believe that especially in the Somali culture khat intake cannot be compared to any food intake, of which the retrospective assessment is problematic. In the Somali culture khat has a special significance, which also comes from existing traditional knowledge about its dangers. Therefore we suppose that retrospective assessment of khat intake is more valid than that of food intake. Also, the effects of social desirability might have affected both the detection of positive screening cases and the assessment of khat use. The fact that dozens of families approached the compound of our collaborating partner agencies in order to find help for their mentally ill members (often they brought them in chains to the compound) shows the great need for mental health services. Thus, the tendency to hide family members from our interviewer teams can be estimated as marginal. On the contrary, during the interviews our greater fear was that households would over-report the presence of mentally challenged family members. For this reason, we counted only those persons as positive screening cases when their existence in the household could be validated (e.g. by their physical presence). The assessment of khat consumption is another point where effects of social desirability can be assumed. Whereas the information given by examined cases could be validated by the information of family members, we were well aware of the danger that the matched controls might have under-reported their khat intake. In order to allow for this in the interview we spent much time and effort to identify the real khat intake (e.g. often we had to 'negotiate' about the answer to the khat questions – a habit inherent in Somali culture). Nevertheless, the high correlations validate the assessment procedures.

Also, the sample size was too small to answer some of the questions in detail, especially the small number of female examined cases. However, the results were nonetheless statistically very robust.

In sum, our design could not determine the existence of a causal relationship between khat and psychosis – this would have been too ambitious a goal. However, our main findings do not contradict the hypothesis that khat might cause psychosis.

Future research should focus on the question of causality. Unfortunately, the Horn of Africa is probably the region where stimulant abuse currently reaches highest levels on a world-wide scale. This circumstance offers a unique research opportunity for cross-sectional and repeated measurement studies, which would enable us better to understand the relationship between schizophrenia and drug effects and to fill the gap in knowledge related to khat use. At the same time, the alarmingly high prevalence of khat chewing among persons who suffer from psychotic symptoms in one of the countries of its highest use, and the magnitude of human suffering associated with it, demands the development of adequate community-based prevention and treatment interventions. The usefulness of standard procedures derived from developed countries and brought to the Horn of Africa must be viewed in a "service-research attitude"[38]. We believe that khat abuse has become a tragic obstacle for the reconstruction of this war-torn society; consequently, there is an urgent need to address this mental health issue with means other than prohibition and regulation of the demand side through taxation, as khat is integral to the Somali culture.

## Conclusion

Not khat intake per se but rather specific patterns of it are linked to the development of psychotic symptoms, like early onset in life, excessive chewing (> 2 bundles/day) and use as self-medication for war trauma related symptoms. Although we cannot establish a causal relationship between khat and psychosis, we find some evidence that the prevalence of psychotic disorders is increased among the male adult population of Hargeisa. Ex-combatants are the group in society who are most affected by severe mental disorders, among them we find the highest abuse of khat. The way of khat use in Hargeisa is changing, indicated by e.g. the development of a group of heavy users who show consumption patterns similar to amphetamine addicts in Western countries. Measures of prevention and treatment of and further research on khat-related severe mental disorders have to be undertaken, however, taking into consideration that khat is an integral part of the Somali culture, which cannot simply be prohibited.

## Competing interests

The author(s) declare that they have no competing interests.

## Authors' contributions

Development of the study design and selection of instruments were accomplished by MO, FN, TE, MS, BR and HeH. MS, TE and MO performed pilot studies. Training and supervision of local interviewers was provided by MO, HaH, FN and BL. Clinical interviews were conducted by MO, MS, CC and BL. Statistical analysis was performed by MO and TE. The article was composed and revised by MO, TE, BR, FN and HeH.

## Pre-publication history

The pre-publication history for this paper can be accessed here:


